# Recommendations for Medical Management of Adult Lead Exposure

**DOI:** 10.1289/ehp.9784

**Published:** 2006-12-22

**Authors:** Michael J. Kosnett, Richard P. Wedeen, Stephen J. Rothenberg, Karen L. Hipkins, Barbara L. Materna, Brian S. Schwartz, Howard Hu, Alan Woolf

**Affiliations:** 1 Division of Clinical Pharmacology and Toxicology, Department of Medicine, University of Colorado Health Sciences Center, Denver, Colorado, USA; 2 Department of Veterans Affairs, New Jersey Health Care System, East Orange, New Jersey, USA; 3 CINVESTAV-IPN (Centro de Investigaciones y de Estudios Avanzados-Instituto Politécnico Nacional), Merida, Yucatan, Mexico; 4 National Institute of Public Health, Cuernavaca, Morelos, Mexico; 5 Public Health Institute, Occupational Lead Poisoning Prevention Program, Richmond, California, USA; 6 California Department of Health Services, Occupational Health Branch, Richmond, California, USA; 7 Departments of Environmental Health Sciences and Epidemiology, Johns Hopkins Bloomberg School of Public Health, Baltimore, Maryland, USA; 8 Department of Medicine, Johns Hopkins School of Medicine, Baltimore, Maryland, USA; 9 Department of Environmental Health Sciences, University of Michigan School of Public Health, Ann Arbor, Michigan, USA; 10 Program in Environmental Health, Division of General Pediatrics, Children’s Hospital, Boston, Massachusetts, USA

**Keywords:** adult lead exposure, blood lead, chelation, medical management, medical surveillance, pregnancy

## Abstract

Research conducted in recent years has increased public health concern about the toxicity of lead at low dose and has supported a reappraisal of the levels of lead exposure that may be safely tolerated in the workplace. In this article, which appears as part of a mini-monograph on adult lead exposure, we summarize a body of published literature that establishes the potential for hypertension, effects on renal function, cognitive dysfunction, and adverse female reproductive outcome in adults with whole-blood lead concentrations < 40 μg/dL. Based on this literature, and our collective experience in evaluating lead-exposed adults, we recommend that individuals be removed from occupational lead exposure if a single blood lead concentration exceeds 30 μg/dL or if two successive blood lead concentrations measured over a 4-week interval are ≥ 20 μg/dL. Removal of individuals from lead exposure should be considered to avoid long-term risk to health if exposure control measures over an extended period do not decrease blood lead concentrations to < 10 μg/dL or if selected medical conditions exist that would increase the risk of continued exposure. Recommended medical surveillance for all lead-exposed workers should include quarterly blood lead measurements for individuals with blood lead concentrations between 10 and 19 μg/dL, and semiannual blood lead measurements when sustained blood lead concentrations are < 10 μg/dL. It is advisable for pregnant women to avoid occupational or avocational lead exposure that would result in blood lead concentrations > 5 μg/dL. Chelation may have an adjunctive role in the medical management of highly exposed adults with symptomatic lead intoxication but is not recommended for asymptomatic individuals with low blood lead concentrations.

As a likely consequence of its capacity to interfere with biochemical events present in cells throughout the body, inorganic lead exerts a wide spectrum of multisystemic adverse effects. These health impacts range from subtle, subclinical changes in function to symptomatic, life-threatening intoxication. In recent years, research conducted on lead-exposed adults has increased public health concern over the toxicity of lead at low dose. These findings support a reappraisal of the levels of lead exposure, sustained for either short or extended periods of time, that may be safely tolerated in the workplace. In this article we offer health-based recommendations on the management of lead-exposed adults aimed at primary and secondary prevention of lead-associated health problems. As noted in the introduction to this mini-monograph (Schwartz and Hu 2007) the authors of this article are an independent subgroup of an expert panel (8 of 13 members) originally convened by the Association of Occupational and Environmental Clinics (www.aoec.org) to address these management issues. In deriving the recommendations in this article, we took note of a body of literature that establishes the potential for adverse health effects at blood lead concentrations or exposure levels permissible under current workplace regulations established in the 1970s by the U.S. Occupational Safety and Health Administration (OSHA). These regulations generally require removal from lead exposure when whole-blood lead concentrations exceed 50 or 60 μg/dL. These values are considerably above blood lead concentrations of the general population of the United States, which had a geometric mean of 12.8 μg/dL in the late 1970s (National Center for Health Statistics 1984), and a recent value of 1.45 μg/dL [U.S. Centers for Disease Control and Prevention (CDC) 2005].

In setting forth our perspective on the recommended medical management of adult lead exposure, the narrative of this article focuses on four categories of health effects—hypertension, decrement in renal function, cognitive dysfunction, and adverse reproductive outcome—that have been the subject of much recent research. The discussion of these end points highlights those studies, that by virtue of their design and scope, were particularly influential in establishing the authors’ concerns regarding the potential for adverse health effects at low to moderate levels of lead exposure in adults. Collectively, these effects support the preventive medical management strategies that are recommended in the tables. A review of the extensive literature on the health effects of lead is beyond the scope of this article, but the reader is referred to reviews on the cardiovascular and cognitive impacts of lead on adults that appear elsewhere in this mini-monograph (Navas-Acien et al. 2007; Shih et al. 2007), as well as a review on recent lead literature prepared by the U.S. Environmental Protection Agency (EPA) for its Air Quality Criteria for Lead (U.S. EPA 2006).

Table 1 is a summary of the adverse health risks associated with different blood lead concentrations and presents corresponding medical management recommendations that range from discussion of risks and reduction of lead exposure at low levels to removal from lead exposure accompanied by probable chelation therapy at the highest levels. The designation of risks as either “short-term” or “long-term,” depending on whether the risks are associated with exposure lasting less than or more than 1 year, reflects a qualitative understanding of the duration of lead exposure that may be required to elicit certain adverse health effects of lead. For some of the long-term risks, such as hypertension, research employing noninvasive K-shell X-ray fluorescence measurement of lead in bone, a biomarker of long-term cumulative exposure, suggests that several years of sustained elevations in blood lead may be necessary for a significant risk to emerge. The use of 1 year as a cut-point in the table is not intended to represent a sharp division, in terms of cumulative dose, between what might constitute a short-term versus a long-term risk nor does it imply that a significant long-term risk begins to exist as soon as 1 year is surpassed. Blood lead, a measure of the amount of lead circulating in the tissues, reflects both recent exogenous exposure as well as endogenous redistribution of lead stored in bone.

The categorization of risks in Table 1 by discrete intervals of blood lead concentration is a qualitative assessment. In clinical practice, substantial interindividual variability in the susceptibility to symptomatic adverse effects of lead is commonly observed. Factors that might influence the risk of lead toxicity in adults include preexisting disease affecting relevant target organs (e.g., hypertension, renal disease, or neurologic dysfunction), nutritional deficiencies that modify the absorption or distribution of lead (e.g., low dietary calcium or iron deficiency), advanced age, and genetic susceptibility. Although recent studies suggest that polymorphisms in specific genes may modify the toxicokinetics and renal effects of lead (Theppeang et al. 2004; Weaver et al. 2006; Wu et al. 2003), research findings at present are insufficient to conclusively identify genotypes that confer increased risk.

## Health Effects at Low Dose

### Hypertension

Animal investigations support a pressor effect of lead at low dose (Fine et al. 1988; Gonick et al. 1997; Vaziri 2002). Epidemiologic investigations conducted in large general population samples (e.g., Harlan 1988; Nash et al. 2003; Pocock et al. 1988; Schwartz 1988) suggest lead may elevate blood pressure in adults at blood lead concentrations < 20 μg/dL. In some human studies of the link between blood lead and blood pressure, the relationship appeared to be influenced by subjects’ sex or race (e.g., Den Hond et al. 2002; Staessen et al. 1996; Vupputuri et al. 2003). Three meta-analyses of studies examining the relationship between blood lead and blood pressure found relatively consistent effects of blood lead on blood pressure. The studies showed statistically significant coefficients for a 2-fold increase in blood lead of 1.0 mmHg (Nawrot et al. 2002; Staessen et al. 1994) or 1.25 mmHg (Schwartz 1995) for systolic blood pressure, and 0.6 mmHg for diastolic blood pressure (Nawrot et al. 2002; Staessen et al. 1994). The study populations analyzed in these meta-analyses included many with blood lead concentrations < 20 μg/dL.

Further support for the impact of low-level lead exposure on blood pressure has emerged from studies employing K-shell X-ray fluorescence measurement of lead in bone, a biomarker of long-term cumulative lead exposure. In two major studies drawn from samples of the general population, bone lead concentration was a significant predictor of the risk of hypertension (Hu et al. 1996; Korrick et al. 1999). Findings from the study by Hu et al. (1996) illustrate the associated risk. In that general population sample of middle-aged to elderly men (*n* = 590), the average blood lead concentration was 6.3 μg/dL. On the basis of the subjects’ ages (mean 67 ± 7.2 years), it may be expected that they lived most of their adult lives at a time when the blood lead concentration of the general population ranged from 10 to 25 μg/dL (Hofreuter et al. 1961; Mahaffey et al. 1982; Minot 1938). Comparing the lowest with the highest quintile of bone lead among that cohort, a tibia bone lead increment of 29 μg/g was associated with a 1.5 odds ratio (OR) for hypertension [95% confidence interval (CI), 1.1–1.8]. Given the slope of 0.05 that has described the linear relationship between tibia bone lead concentration and cumulative blood lead index in subjects with chronic lead exposure in many studies (Hu et al. 2007), this increment in bone lead is roughly equivalent to a cumulative blood lead index of 580 μg/dL · years (i.e., 29 μ 0.05 = 580). Considered in the context of a 40-year working lifetime, the risk of lead-associated hypertension may be significantly reduced by preventive measures that lower chronic workplace blood lead concentrations from the 20s and 30s μg/dL range to < 10 μg/dL. For example, a change in average workplace blood lead concentration from 25 to 10 μg/dL over a 40-year working lifetime would reduce a worker’s cumulative blood lead index by 600 μg/dL · years, slightly more than the 580 μg/dL · years cited above.

Hypertension is a significant risk factor for cardiovascular and cerebrovascular mortality. As reviewed in an accompanying article in this mini-monograph (Navas-Acien et al. 2007), studies conducted in general population cohorts have consistently observed a positive association between lead exposure and cardiovascular disease. Because of their size and design, studies derived from the National Health and Nutrition Evaluation Surveys (NHANES) are particularly notable. A 16-year longitudinal analysis of the general population cohort studied between 1976 and 1980 as part of NHANES II found that blood lead concentrations of 20–29 μg/dL at baseline were associated with 39% increased mortality from circulatory system disease compared with subjects with blood lead < 10 μg/dL [relative risk (RR) 1.39; 95% CI, 1.01–1.91] (Lustberg and Silbergeld 2002). Two studies recently examined the longitudinal relationship between blood lead concentration and cardiovascular mortality among participants in NHANES III. In a 12-year longitudinal study of participants in NHANES III, ≥ 40 years of age (*n* = 9,757), the subgroup with blood lead concentration ≥ 10 μg/dL (median, 11.8) had a relative risk of cardiovascular mortality of 1.59 (95% CI, 1.28–1.98) compared with subjects with blood lead < 5 μg/dL (Schober et al. 2006). In a 12-year longitudinal analysis of subjects ≥ 17 years of age (*n* = 13,946), the relative risk for cardiovascular mortality was 1.53 (95% CI, 1.21–1.94), comparing a blood lead of 4.92 μg/dL (80th percentile of the distribution) with a blood lead of 1.46 μg/dL (20th percentile of the distribution) (Menke et al. 2006).

### Renal effects

Renal injury that appears after acute high-dose lead exposure may include reversible deficits in proximal tubular reabsorption and prerenal azotemia induced by renal vasoconstriction and/or volume depletion (Coyle et al. 200; Wedeen et al. 1979). In a minority of exposed individuals, years of chronic, high-dose lead exposure may result in chronic lead nephropathy, a slowly progressive interstitial fibrosis characterized by scant proteinuria (Lilis et al. 1968). Epidemiologic investigations of renal function in workers with lower levels of chronic lead exposure have yielded variable findings. For example, in a cohort of approximately 800 current and former lead workers with mean blood lead of 32 ± 15 μg/dL, there was no significant linear relationship between blood lead concentration and two measures of renal function, serum creatinine and creatinine clearance (Weaver et al. 2003). There was an interaction between age and tibia lead concentration, a biomarker of cumulative lead exposure, on these same biomarkers, resulting in a trend toward worse renal function with increasing bone lead in the oldest tercile of workers (> 46 years of age) but improved renal function with increasing bone lead in the youngest workers (≤ 36 years of age). The authors suggested that lead-induced hyperfiltration, a finding noted in other studies, might presage the eventual development of lead-induced renal insufficiency. Both blood lead and tibia lead were correlated with increased urinary *N*-acetyl-β-d-glucosaminidase (NAG), a biomarker of early biological effect on the renal tubule, but in an analysis of a smaller subset of the lead workers (*n* = 190) that controlled for the relatively low levels of urinary cadmium (1.1 ± 0.78 μg/g creatinine), only the relationship with tibia lead and NAG remained significant (Weaver et al. 2003). Among a cohort of 70 active lead workers with a median blood lead concentration of 32 μg/dL (range, 5–47), there were modest correlations between blood lead and urinary β-2-microglobulin (*r* = 0.27; *p* = 0.02), and between cumulative blood lead index and NAG (*r* = 0.25; *p* = 0.04) (Gerhardsson et al. 1992).

Several studies conducted in general population samples have reported an association between blood lead concentration and common biomarkers of renal function (serum creatinine and creatinine clearance). In a cross-sectional investigation of a subcohort of middle-aged to elderly men enrolled in the Normative Aging Study (*n* = 744), there was a negative correlation between blood lead (mean, 8.1 ± 3.9 μg/dL; range, < 4.0–26.0 μg/dL) and measured creatinine clearance, after natural log transformation of both variables and adjustment for other covariates (Payton et al. 1994). Among an adult population that included subjects with environmental cadmium exposure [*n* = 965 men (geometric mean blood lead, 11.4 μg/dL; range, 2.3–72.5 μg/dL); *n* = 1,016 women (geometric mean blood lead, 7.5 μg/dL; range, 1.7–60.3 μg/dL)], log-transformed blood lead concentration was inversely correlated with measured creatinine clearance (Staessen et al. 1992). In a population-based study of Swedish women 50–59 years of age (*n* = 820), low levels of blood lead (mean 2.2 μg/dL; 5th–95th percentiles, 1.1–4.6 μg/dL) were inversely correlated with creatinine clearance and glomerular filtration rate, after adjusting for age, body mass index, urinary or blood cadmium, hypertension, diabetes, and regular use of nonsteroidal anti-inflammatory drug (NSAID) medication (Akesson et al. 2005).

Individuals with other risk factors for renal disease, notably hypertension and diabetes, may be more susceptible to an adverse impact of low-level lead exposure on renal function. Among adults participating in NHANES III (*n* = 15,211), blood lead was a risk factor for elevated serum creatinine (defined as ≥ 99th percentile of the analyte’s race and sex specific distributions, generally > 1.2–1.5 mg/dL) and “chronic kidney disease” (defined as an estimated glomerular filtration rate < 60 mL/min) only among subjects with hypertension (*n* = 4813) (Muntner et al. 2003). Compared with hypertensives in the lowest quartile of blood lead (range, 0.7–2.4 μg/dL), hypertensive subjects in the next highest quartile of blood lead (range, 2.5–3.8 μg/dL) had a covariate adjusted OR for elevated serum creatinine of 1.47 (95% CI, 1.03–2.10) and for chronic kidney disease of 1.44 (95% CI, 1.00–2.09). At the next highest quartile of blood lead (range, 3.9–5.9 μg/dL), the covariate-adjusted OR for elevated serum creatinine was 1.80 (95% CI, 1.34–2.42), and for chronic kidney disease it was 1.85 (95% CI, 1.32–2.59). In a subcohort of middle-aged to elderly men participating in the Normative Aging Study (*n* = 427, blood lead 4.5 ± 2.5 μg/dL), multiple regression analysis revealed that log-transformed blood lead was positively correlated with serum creatinine in hypertensive but not normotensive subjects (Tsaih et al. 2004). In a longitudinal study of this cohort over a mean of 6 years, an interaction between lead and diabetes yielded a positive association between baseline blood lead concentration and change in serum creatinine that was strongest in diabetic subjects (Tsaih et al. 2004). An interaction with diabetes was also present in the association of tibial lead concentration with longitudinal change in serum creatinine (Tsaih et al. 2004). Although these general population studies are consistent with an adverse effect of lead exposure on renal function at notably low levels, the extent to which diminished renal function may itself result in increased body lead burden has not been fully elucidated.

### Cognitive dysfunction

A few studies examining relatively small numbers of workers (*n* ≤ 100) with blood lead concentrations ranging approximately 20–40 μg/dL have associated lead exposure with subclinical decrements in selective domains of neurocognitive function (Barth et al. 2002; Hänninen et al. 1998; Mantere et al. 1984; Stollery 1996). Among a large cohort of current and former inorganic lead workers studied in Korea, a cross-sectional analysis (*n* = 803 workers) (Schwartz et al. 2001) and a 3-year longitudinal analysis (*n* = 576 workers) (Schwartz et al. 2005) found that blood lead concentrations across the approximate range of 20–50 μg/dL were associated with subclinical neurocognitive deficits. Among a small population of former lead workers (*n* = 48) and age-matched controls with similar blood lead concentrations (approximately 5 μg/dL in both groups; range, 1.6–14.5 μg/dL; mean age, 39.8 years), increases in current blood lead concentration within the entire study population were correlated with poorer performance on several tests of neurocognitive function but on only one measure was cumulative lead exposure (measured in the workers) associated with poorer performance (Winker et al. 2005).

In the population-based sample of adults 20–59 years of age participating in the NHANES III study (*n* = 4937), there was no relationship between blood lead concentration (geometric mean, 2.51 μg/dL) and covariate-adjusted performance on neurocogntive function (Krieg et al. 2005). However, significant associations have emerged in some studies of older adults with slightly higher blood lead concentrations. In a rural subset of elderly women (mean age, 71.1 ± 4.7 years; *n* = 325) with background, community lead exposure (geometric mean blood lead concentration, 4.8 μg/dL; range, 1–21 μg/dL), certain measures of neuropsychologic function (Trailmaking part B and Digit Symbol test) were performed more poorly by women in the upper 15th percentile of blood lead (blood lead ≥ 8 μg/dL, *n* = 38; Muldoon et al. 1996). However, in the slightly younger subset of elderly women who resided in an urban area (mean age, 69.4 ± 3.8 years; *n* = 205), no relationship between blood lead (geometric mean, 5.4 μg/dL) and neuropsychologic performance was discernible (Muldoon et al. 1996). In a general population sample of middle-aged to elderly men (*n* = 141; mean age, 66.8 ± 6.8 years) with a mean blood lead concentration of 5.5 ± 3.5 μg/dL examined as part of the Normative Aging Study, increased blood lead concentration was associated with poorer performance on neuropsychologic assessment of memory, verbal ability, and mental processing speed (Payton et al. 1998). In a larger subset of men (*n* = 736; mean age, 68.2 ± 6.9 years) from the Normative Aging Study assessed with the Mini-Mental Status Examination (MMSE), the OR for having a test score associated with an increased risk of dementia was 3.4 (95% CI, 1.6–7.2) comparing the mean blood lead of the highest quartile (mean, 8.9 μg/dL) to that of the lowest quartile (mean, 2.5 μg/dL) (Wright et al. 2003). There was a positive interaction between age and blood lead, which is consistent with a lead-associated acceleration in age-related neurodegeneration.

As reviewed in an accompanying article in this mini-monograph (Shih et al. 2007), there is evidence that at low levels of lead exposure, biomarkers of cumulative lead exposure, such as lead in bone, may be associated with an adverse impact on neurocognitive function that is not reflected by measurement of lead in blood. Among subjects from the Normative Aging Study (*n* = 466; mean age, 67.4 ± 6.6 years) examined for longitudinal change in MMSE score over an average of 3.5 ± 1.1 years, higher patella bone lead concentrations, a biomarker of cumulative lead exposure, predicted a steeper decline in performance (Weisskopf et al. 2004). By comparison, baseline blood lead concentration (median, 4 μg/dL; interquartile range = 3, 5) did not predict change in MMSE score. In a longitudinal analysis of performance on a battery of cognitive tests in a subset of the Normative Aging Study, bone lead measurements were predictive of worsening performance over time on tests of visuospatilal/visuomotor ability (Weisskopf et al. 2007). In a cross-sectional analysis of 985 community dwelling residents 50–70 years of age, increasing tibia bone lead concentrations were significantly associated with decrements in cognitive function, whereas an impact of blood lead (mean, 3.46 ± 2.23 μg/dL) was not apparent (Shih et al. 2006).

### Reproductive outcome in women

Adverse effects on reproductive outcome constitute a special risk of lead exposure to women of reproductive age. A nested case–control study examined the association of blood lead concentration with spontaneous abortion in a cohort of 668 pregnant women seeking pre-natal care in Mexico City (Borja-Aburto et al. 1999). After matching for maternal age, education, gestational age at study entry, and other covariates, the OR for spontaneous abortion before 21 weeks gestation was 1.13 (95% CI, 1.01–1.30) for every 1 μg/dL increase in blood lead across the blood lead range of 1.4–29 μg/dL. Compared with the reference category of < 5 μg/dL of blood lead, women whose blood lead levels were 5–9, 10–14, and > 15 μg/dL had ORs for spontaneous abortion of 2.3, 5.4, and 12.2, respectively (test for trend, *p* = 0.03). Although several earlier studies failed to detect this substantial impact, they may have been subject to methodologic limitations not present in the Mexico City investigation (Hertz-Picciotto 2000).

Several studies have found that lead exposure during pregnancy affects child physical development measured during the neonatal period and early childhood. In an extensively studied cohort of 272 full-term, parturient women from Mexico City with environmental lead exposure common to the region (mean maternal blood lead, 8.9 ± 4.1 μg/dL; mean tibia bone lead, 9.8 ± 8.9 μg/g; range, 12–38 μg/g), every increase of 10 μg/g in maternal tibia lead was associated with a 73-g (95% CI, 25–121) decrease in birth weight (Gonzalez-Cossio et al. 1997). The impact of tibia bone lead on birth weight was nonlinear and was most pronounced in mothers with the highest quartile of bone lead (> 15–38 μg/g) where the decrement relative to the lowest quartile was estimated to be 156 g. Primarily in the same cohort, a maternal patella lead concentration > 24.7 μg/g was associated with an OR of 2.35 (95% CI, 1.26–4.40) for a neonate with one category smaller head circumference at birth, assessed as a five-category–ordered variable (Hernandez-Avila et al. 2002). In a different Mexico City cohort, each doubling of maternal blood lead at 36 weeks of pregnancy (geometric mean, 8.1 μg/dL; 25th–75th percentile, 5–12 μg/dL) was associated with a decrease of 0.37 cm (95% CI, 0.57–0.17) in the head circumference of a 6-month-old infant (Rothenberg et al. 1999b).

Prenatal lead exposure assessed by umbilical cord blood lead concentration has been inconsistently associated with an adverse effect on neurobehavioral development in childhood. However, recent studies suggest that mobilization of maternal bone lead during pregnancy may contribute to fetal lead exposure in ways that may be incompletely reflected by the single measurement of umbilical cord whole-blood lead (Chuang et al. 2001; Tellez-Rojo et al. 2004). In a prospective study conducted in Mexico City of 197 mother–infant pairs, a statistically significant adverse effect of umbilical cord blood lead (mean, 6.7 ± 3.4 μg/dL; range, 1.2–21.6 μg/dL) was also accompanied by an independent adverse effect of maternal bone lead burden on the 24-month Mental Development Index (MDI) of the Bayley Scales of Infant Development, which decreased 1.6 points (95% CI, 0.2–3.0) for every 10-μg/g increase in maternal patellar lead (mean, 17.9 ± 15.2 μg/g; range, <1–76.6 μg/g) (Gomaa et al. 2002).

A prospective study that measured maternal plasma lead and maternal whole-blood lead during pregnancy found that maternal plasma lead during the first trimester was the stronger predictor of infant mental development at 24 months of age (Hu et al. 2006). In this cohort, first trimester maternal plasma lead was 0.016 ± 0.014 μg/dL and first trimester maternal whole-blood lead was 7.07 ± 5.10 μg/dL (*n* = 119). Adjusting for covariates that included maternal age, maternal IQ, child sex, childhood weight and height for age, and childhood whole-blood lead at 24 months, an increase of one SD in log_e_ (natural log)–transformed plasma lead in the first trimester was associated with a 3.5-point decrease in score on the 24-month MDI of the Bayley Scales of Infant Development. The corresponding impact of one SD increase in log_e_ maternal whole blood during the first trimester was a 2.4-point decrease in the 24-month MDI. The logarithmic relationship between maternal plasma and blood lead concentrations and infant MDI indicated that the strongest effects occurred among mothers with the lowest plasma and blood lead concentrations.

Two long-term prospective studies that conducted multiple measurements of maternal blood lead during pregnancy and childhood have identified an adverse impact of low-level prenatal lead exposure on postnatal neurobehavioral development extending beyond infancy. Applying a repeated measures linear regression technique to analysis of age-appropriate IQ test data obtained in 390 children 3–7 years of age, the Yugoslavia Prospective Lead Study found independent adverse effects of both prenatal and postnatal blood lead. After controlling for the pattern of change in postnatal blood lead and other covariates, IQ decreased 1.8 points (95% CI, 1.0–2.6) for every doubling of prenatal blood lead, which was assessed as the average of maternal blood lead at midpregnancy and delivery (mean, 10.2 ± 14.4 μg/dL; *n* = 390) (Wasserman et al. 2000). The Mexico City Prospective Lead Study used generalized linear mixed models with random intercept and slope to assess the impact on IQ measured at 6–10 years of age of blood lead measurements systematically obtained during weeks 12, 20, 24, and 36 of pregnancy, at delivery, and at multiple points throughout childhood (Schnaas et al. 2006). Geometric mean blood lead during pregnancy was 8.0 μg/dL (range, 1–33 μg/dL; *n* = 150); from 1 through 5 years it was 9.8 μg/dL (2.8–36.4 μg/dL), and from 6 through 10 years it was 6.2 μg/dL (range, 2.2–18.6 μg/dL). IQ at 6 to 10 years of age, assessed by the Wechsler Intelligence Scale for Children—Revised, decreased significantly only with increasing natural-log third-trimester blood lead, controlling for other blood lead measurements and covariates. Every doubling of third trimester blood lead (geometric mean of maternal blood lead at weeks 28 and 36 = 7.8 μg/dL, 5th–95th percentile: range, 2.5–24.6 μg/dL) was associated with an IQ decrement of 2.7 points (95% CI, 0.9–4.4). Notably, the nonlinear (i.e., log-linear) relationships detected in the Yugoslavia and Mexico City studies indicate that across a maternal blood lead range of 1–30 μg/dL, an increase in blood lead from 1 to 10 μg/dL will account for more than half the IQ decrement.

Two independent cohorts have provided evidence that maternal lead burden during pregnancy may be associated with increased risk of pregnancy hypertension and/or elevated blood pressure during pregnancy. In a retrospective study of 3,210 women during labor and delivery, increasing umbilical cord blood lead levels (mean, 6.9 ± 3.3 μg/dL; range, 0–35 μg/dL) were associated with increased systolic blood pressure during labor (1.0 mmHg for every doubling of blood lead) and increased odds of hypertension (not further defined) recorded any time during pregnancy (OR = 1.3; 95% CI, 1.1–1.5) for every doubling of blood lead (Rabinowitz et al. 1987). A prospective study of third trimester blood lead (geometric mean, 2.3 ± 1.4 μg/dL; range, 0.5–36.5 μg/dL) in 1,188 predominantly Latina immigrants showed that, in the immigrants, every doubling in blood lead was associated with increased third-trimester systolic blood pressure (1.2 mmHg; 95% CI, 0.5–1.9) and diastolic blood pressure (1.0 mmHg; 95% CI, 0.4–1.5) (Rothenberg et al. 1999a). A study of a subset of the same cohort (*n* = 637) without regard to immigration status found that every 10-μg/g increase in calcaneus (heel) bone lead increased the OR of third trimester pregnancy hypertension (systolic > 90 and/or diastolic > 140 mmHg) by 1.86 (95% CI, 1.04–3.32) (Rothenberg et al. 2002).

## Medical Surveillance for Lead-Exposed Workers

The OSHA workplace standard for lead exposure in general industry (adopted in 1978) and a corresponding standard for lead exposure in construction trades (adopted in 1993) set forth medical surveillance requirements that include baseline and periodic medical examinations and laboratory testing. Details of the two standards, which establish distinct criteria for the implementation of surveillance, can be found on the OSHA website (OSHA 2002). Because of the concern regarding adverse health effects of lead associated with the lower levels of exposure discussed in this article, we recommend a revised schedule of medical surveillance activities (Table 2). Unlike the OSHA medical surveillance requirements, which apply only to workers exposed to airborne lead levels ≥ 30 μg/m^3^ as an 8-hr time-weighted average, the recommendations in Table 2 are intended to apply to all lead-exposed workers who have the potential to be exposed by lead ingestion, even in the absence of documented elevations in air lead levels (Sen et al. 2002). As shown in Table 2, the level of a worker’s current blood lead measurement, as well as possible changes in lead-related exposure, influences the recommended time interval for subsequent blood lead measurements. Blood lead measurements should be obtained from a clinical laboratory that has been designated by OSHA as meeting the specific proficiency requirements of the OSHA lead standards. OSHA maintains a list of these laboratories on its website (OSHA 2005). Venous blood should be used for biological monitoring of adult lead exposures, except where prohibited by medical or other reasons. Routine measurement of zinc protoporphyrin, a requirement of the OSHA lead standards, is not recommended in Table 2 because it is an insensitive biomarker of lead exposures in individuals with blood lead concentrations <25 μg/dL (Parsons et al. 1991).

The content of the baseline or preplacement history and physical examination for lead-exposed workers should continue to follow the comprehensive scope set forth in the OSHA lead standard for general industry. Measurement of serum creatinine will identify individuals with chronic renal dysfunction who may be subject to increased health risks from lead exposure. With the potential exception of an annual blood pressure measurement and a brief questionnaire regarding the presence of medical conditions (such as renal insufficiency) that might increase the risk of adverse health effects of lead exposure, medical evaluations for lead-exposed workers should be unnecessary as long as blood lead concentrations are maintained < 20 μg/dL. Annual education of lead workers regarding the nature and control of lead hazards, and ongoing access to health counseling regarding lead-related health risks are recommended as preventive measures.

## Lead Exposure during Pregnancy and Lactation

As summarized earlier in this article, the recent findings concerning lead-related adverse reproductive outcomes render it advisable for pregnant women to avoid occupational or avocational lead exposure that would result in blood lead concentrations > 5 μg/dL. Calcium supplementation during pregnancy may be especially important for women with past exposure to lead. Calcium decreases bone resorption during pregnancy (Janakiraman et al. 2003) and may minimize release of lead from bone stores and subsequent fetal lead exposure (Gomaa et al. 2002).

Maternal body lead burden and external lead exposure influence the lead concentration of breast milk (Ettinger et al. 2006; Gulson et al. 1998). The few studies that used ultraclean techniques and mass spectrometry analyses report human breast milk concentrations ranging from 0.6 to 3% of maternal blood lead (Ettinger et al. 2004b; Gulson et al. 1998; Manton et al. 2000; Sowers et al. 2002). Using 1% as a guide, it can be estimated that nursing mothers with a blood lead concentration < 20 μg/dL will have breast milk with a concentration < 2 μg/L, a value that approximates the amount of lead in infant formula (Gulson et al. 2001). A recent randomized clinical trial among Mexican women with mean blood lead concentrations of approximately 9 μg/dL found that calcium supplementation during lactation may reduce the lead concentration of breast milk by 5–10% (Ettinger et al. 2006). Breast feeding should be encouraged for almost all women (Ettinger et al. 2004a; Sanin et al. 2001; Sinks and Jackson 1999), with decisions concerning women with very high lead exposure addressed on an individual basis.

## Medical Treatment of Elevated Blood Lead Concentration and Overt Lead Intoxication

Removal from all sources of hazardous lead exposure, whether occupational or nonoccupational, constitutes the first and most fundamental step in the treatment of an individual with an elevated blood lead concentration. A careful history that inquires about a broad spectrum of potential lead sources is recommended (Occupational Lead Poisoning Prevention Program 2006). Removal from occupational lead exposure will usually require transfer of the individual out of any environment or task that might be expected to raise the blood lead concentration of a person not using personal protective equipment above background levels (i.e., 5 μg/dL). If there has been a history of an affected individual bringing lead-contaminated shoes, work clothes, or equipment home from the workplace, evaluation of vehicles and the home environment for significant levels of lead-containing dust might be considered (Piacitelli et al. 1995). Although such “take-home” exposure might contribute to further lead exposure of the worker, it ordinarily poses more of a potential risk to young children and pregnant or nursing women who share the worker’s home environment (Hipkins et al. 2004; Roscoe et al. 1999).

Medical treatment of individuals with overt lead intoxication involves decontamination, supportive care, and judicious use of chelating agents. Comprehensive discussion of such treatment is beyond the scope of this article but has been reviewed in recent medical toxicology texts (Kosnett 2001, 2005). A variety of chelating agents has been demonstrated to decrease blood lead concentrations and increase urinary lead excretion. A recent double-blind randomized clinical trial of oral chelation in young children with blood lead concentrations ranging from 22 to 44 μg/dL found that the drug succimer lowered blood concentrations transiently but did not improve cognitive function (Dietrich et al. 2004; Rogan et al. 2001). Although anecdotal evidence suggests that chelation has been associated with improvement in symptoms and decreased mortality in patients with lead encephalopathy, controlled clinical trials demonstrating efficacy are lacking. Treatment recommendations are therefore mostly empiric, and decisions regarding the initiation of chelation therapy for lead intoxication have occasionally engendered controversy.

In our experience, adults with blood lead concentrations ≥ 100 μg/dL almost always warrant chelation, as levels of this magnitude are often associated with significant symptoms and may be associated with an incipient risk of encephalopathy or seizures. Occasionally, patients with very high blood lead concentrations may have no overt symptoms. Patients with blood lead concentrations of 80–99 μg/dL, with or without symptoms, can be considered for chelation treatment, as may some symptomatic individuals with blood lead concentrations of 50–79 μg/dL. These demarcations are imprecise, however, and decisions on chelation should be made on a case-by-case basis after consultation with an experienced specialist in occupational medicine or medical toxicology.

Hair lead analysis or measurement of urine lead concentration seldom provide exposure information of clinical value beyond that provided by the history and the measurement of blood lead concentration. Chelation initiated exclusively on the basis of hair or urine lead levels or chelation of asymptomatic individuals with low blood lead concentrations is not recommended.

Adults with overt lead intoxication will generally experience improvement in symptoms after removal from lead exposure and decline in blood lead concentration. This clinical observation on improvement in overt symptoms finds some support from the relatively limited number of studies that have examined the impact of naturally declining blood lead concentrations on cognitive function in occupationally exposed subjects (Chuang et al. 2005; Lindgren et al. 2003; Winker et al. 2006). Improvement or resolution of neurocognitive or neurobehavioral symptoms may sometimes lag the decline in blood lead concentration, possibly because of the relatively slower removal of lead from the central nervous system (Cremin et al. 1999; Goldstein et al. 1974). The pace of improvement can be highly variable, and may range from weeks to a year or more depending on the magnitude of intoxication. Anecdotal experience and analogy to other forms of brain injury suggest a potential role for rehabilitative services (e.g., physical therapy, cognitive rehabilitation) in enhancing the prospect for recovery, and in demonstrating the capacity for safe return to work. Short-term improvement in neurocognitive function associated with a decline in blood lead concentration does not obviate concern that long-term cumulative lead exposure may nonetheless have a deleterious effect on cognitive reserve, and may accelerate age-related decline in cognitive function (Schwartz et al. 2005; Weisskopf et al. 2004).

## Additional Management Considerations

With appropriate engineering controls, safe work practices, and personal protective equipment, workers without a previous history of substantial lead exposure should be able to work with lead in a manner that minimizes the potential for hazardous levels of exposure. For such workers, elevations in blood lead concentration that result from unforeseen transient increases in exposure will often decline promptly once the exposure is controlled. However, in a worker with a long history of high exposure, redistribution of lead from a large internal skeletal burden may result in a prolonged elevation of blood lead concentration despite marked reductions in external lead dose.

The recommendations for management of adult lead exposure contained in this article are derived from consideration of risks to health, and have not been the subject of a cost-benefit analysis examining economic feasibility or social impacts. Nonmedical, socioeconomic factors will likely influence how workers, employers, and clinicians respond to the recommendations. In particular, the blood lead concentrations for which some major interventions, such as removal from lead exposure, are recommended are considerably lower than those explicitly specified in the current OSHA lead standards (OSHA 2002). The OSHA standards do require an employer to implement reductions in exposure recommended by a physician who determines an employee has a “detected medical condition” that places him or her at increased risk of “material impairment to health.” This nonspecific provision could form the basis for implementation of protective workplace action at the lower blood concentrations recommended by the authors. Nonetheless, clinicians should inform patients that such recommendations may be contested by an employer or an insurer, and could potentially jeopardize their job benefits or work status. Prudent case management that considers the worker’s perspective on their unique health risks and employment situation will usually be advisable.

## Interpretative Guidance for Clinical Laboratory Report Forms

Clinical laboratories routinely offer brief interpretative guidance on the forms that report the result of blood lead concentrations. There is considerable variability among laboratories regarding the content of such guidance, and laboratories exercise their own discretion regarding the source and detail of the information they provide. Unlike the management guidance chart for childhood blood lead concentrations published by the CDC (2002), which is often reproduced by clinical laboratories, no corresponding CDC guidance exists for blood lead concentrations measured in adults. Notwithstanding the limitations inherent in an abbreviated tabular format, Table 3 represents a guidance chart for adult blood lead measurements that is proposed for use by clinical laboratories.

## Figures and Tables

**Table 1:** Health-based management recommendations for lead-exposed adults.

Blood lead level (μg/dL)	Short-term risks (lead exposure < 1 year)	Long-term risks (lead exposure ≥ 1 year)	Management
< 5	None documented	None documented	None indicated
5–9	Possible spontaneous abortion Possible postnatal developmental delay	Possible spontaneous abortion Possible postnatal developmental delay Possible hypertension and kidney dysfunction	Discuss health risks Reduce lead exposure for women who are or may become pregnant
10–19	Possible spontaneous abortion Possible postnatal developmental delay Reduced birth weight	Possible spontaneous abortion Reduced birth weight Possible postnatal developmental delay Hypertension and kidney dysfunction Possible subclinical neurocognitive deficits	As above for BLL 5–9 μg/dL, plus: Decrease lead exposure Increase biological monitoring Consider removal from lead exposure to avoid long-term risks if exposure control over an extended period does not decrease BLL < 10 μg/dL, or if medical condition present that increases risk with continued exposure^a^
20–29	Possible spontaneous abortion Possible postnatal developmental delay Reduced birth weight	Possible spontaneous abortion Possible postnatal developmental delay Reduced birth weight Hypertension and kidney dysfunction Possible subclinical neurocognitive deficits	Remove from lead exposure if repeat BLL measured in 4 weeks remains ≥ 20 μg/dL
30–39	Spontaneous abortion Possible postnatal developmental delay Reduced birth weight	Spontaneous abortion Reduced birth weight Possible postnatal developmental delay Hypertension and kidney dysfunction Possible neurocognitive deficits Possible nonspecific symptoms^b^	Remove from lead exposure
40–79	Spontaneous abortion Reduced birth weight Possible postnatal developmental delay Nonspecific symptoms^b^ Neurocognitive deficits Sperm abnormalities	Spontaneous abortion Reduced birth weight Possible postnatal developmental delay Nonspecific symptoms^b^ Hypertension Kidney dysfunction/nephropathy Subclinical peripheral neuropathy Neurocognitive deficits Sperm abnormalities Anemia Colic Possible gout	Remove from lead exposure Refer for prompt medical evaluation Consider chelation therapy for BLL >50 μg/dL with significant symptoms or signs of lead toxicity
≥ 80	Spontaneous abortion Reduced birth weight Possible postnatal developmental delay Nonspecific symptoms^b^ Neurocognitive deficits Encephalopathy Sperm abnormalities Anemia Colic	Spontaneous abortion Reduced birth weight Possible postnatal developmental delay Nonspecific symptoms^b^ Hypertension Nephropathy Peripheral neuropathy Neurocognitive deficits Sperm abnormalities Anemia Colic Gout	Remove from lead exposure Refer for immediate/urgent medical evaluation Probable chelation therapy

BLL, blood lead level.

a Medical conditions that may increase the risk of continued exposure include chronic renal dysfunction (serum creatinine > 1.5 mg/dL for men and > 1.3 mg/dL for women, or proteinuria), hypertension, neurologic disorders, and cognitive dysfunction.

b Nonspecific symptoms may include headache, fatigue, sleep disturbance, anorexia, constipation, arthralgia, myalgia, and decreased libido.

**Table 2:** Health-based medical surveillance recommendations for lead-exposed workers.

Category of exposure	Recommendations
All lead-exposed workers^a^	Baseline or preplacement medical history and physical examination, baseline
	BLL, serum creatinine
BLL (μg/dL)
< 10	BLL every month for first 3 months of placement, or upon change in task to higher exposure, then BLL every 6 months
	If BLL increases ≥ 5 μg/dL, evaluate exposure and protective measures.
	Increase monitoring if indicated
	See Table 1 for pregnancy concerns
10–19	As above for BLL < 10 μg/dL, plus:
	BLL every 3 months
	Evaluate exposure, engineering controls, and work practices
	Consider removal (see Table 1)
	Revert to BLL every 6 months after 3 BLLs < 10 μg/dL
≥ 20	Remove from exposure if repeat BLL measured in 4 weeks remains ≥ 20 μg/dL, or if first BLL ≥ 30 μg/dL (see Table 1)
	Monthly BLL testing
	Consider return to lead work after 2 BLLs < 15 μg/dL a month apart, then monitor as above

BLL, blood lead level.

a Lead-exposed means handling or disturbing materials with a significant lead content in a manner that could reasonably be expected to cause potentially harmful exposure through inhalation or ingestion.

**Table 3:** Recommended interpretive guidance for clinical laboratories reporting adult blood lead concentrations.

Blood lead level (μg/dL)	Management recommendations and requirements^a^ for adults
< 5	No action needed
5–9	Discuss health risks Reduce exposure for pregnancy
10–19	Discuss health risks. Decrease exposure. Monitor BLL Remove from exposure for pregnancy, certain medical conditions, long-term risks
20–29	Remove from exposure if repeat BLL in 4 weeks remains ≥ 20 μg/dL
30–79	Remove from exposure. Prompt medical evaluation and consultation advised for BLL > 40 μg/dL OSHA requirements may apply Chelation not indicated unless BLL >50 μg/dL with significant symptoms
≥ 80	Urgent medical evaluation and consultation indicated OSHA requirements may apply Chelation may be indicated if symptomatic and/or BLL ≥100 μg/dL

BLL, blood lead level. Primary management of lead poisoning is source identification and removal from exposure. A single BLL does not reflect cumulative body burden or predict long-term effects.

a Refer to OSHA general industry and construction lead standards for occupational exposure.

## References

[b1-ehp0115-000463] Akesson A, Lundh T, Vahter M, Bjellerup, Lidfeltdt J, Nerbrand C (2005). Tubular and glomerular kidney effects in Swedish women with low environmental cadmium exposure. Environ Health Perspect.

[b2-ehp0115-000463] Barth A, Schaffer AW, Osterode W, Winker R, Konnaris C, Valic E (2002). Reduced cognitive abilities in lead-exposed men. Int Arch Occup Environ Health.

[b3-ehp0115-000463] Borja-Aburto VH, Hertz-Picciotto I, Lopez MR, Farias P, Rios C, Blanco J (1999). Blood lead levels measured prospectively and risk of spontaneous abortion. Am J Epidemiol.

[b4-ehp0115-000463] CDC 2002. Managing Elevated BLLs Among Young Children. Atlanta:Centers for Disease Control and Prevention, National Center for Environmental Health.

[b5-ehp0115-000463] CDC 2005. Third National Report on Human Exposure to Environmental Chemicals. NCEH Publ no 05-0570. Atlanta:Centers for Disease Control and Prevention.

[b6-ehp0115-000463] Chuang HY, Chao KY, Tsai SY (2005). Reversible neurobehavioral performance with reductions in blood lead levels–a prospective study on lead workers. Neurotox Teratol.

[b7-ehp0115-000463] Chuang HY, Schwartz J, Gonzales-Cossio T, Lugo MC, Palazuelos E, Aro A (2001). Interrelations of lead levels in bone, venous blood, and umbilical cord blood with exogenous lead exposure through maternal plasma lead in peripartum women. Environ Health Perspect.

[b8-ehp0115-000463] Coyle P, Kosnett MJ, Hipkins KL (2005). Severe lead poisoning in the plastics industry: a report of three cases. Am J Ind Med.

[b9-ehp0115-000463] Cremin JD, Luck ML, Laughlin NK, Smith DR (1999). Efficacy of succimer chelation for reducing brain lead in a primate model of human lead exposure. Toxicol Appl Pharmacol.

[b10-ehp0115-000463] Den Hond E, Nawrot T, Staessen JA (2002). The relationship between blood pressure and blood lead in NHANES III. National Health and Nutritional Examination Survey. J Hum Hypertens.

[b11-ehp0115-000463] Dietrich KN, Ware JH, Salganik M, Radcliff J, Rogan WJ, Rhoads GG (2004). Effect of chelation therapy on the neuropsychological and behavioral development of lead-exposed children after school entry. Pediatrics.

[b12-ehp0115-000463] Ettinger AS, Tellez-Rojo MM, Amarasiriwardena C, Peterson KE, Schwartz J, Aro A (2006). Influence of maternal bone lead burden and calcium intake on levels of lead in breast milk over the course of lactation. Am J Epidemiol.

[b13-ehp0115-000463] Ettinger AS, Tellez-Rojo MM, Amarasiriwardena C, Bellinger D, Peterson J, Schwartz J, Hu H, Hernandez-Avila M (2004a). Effect of breast milk lead on infant blood lead levels at 1 month of age. Environ Health Perspect.

[b14-ehp0115-000463] Ettinger AS, Tellez-Rojo MM, Amarasiriwardena C, Gonzalez-Cossio T, Peterson KE, Aro A (2004b). Levels of lead in breast milk and their relation to maternal blood and bone lead levels at one-month postpartum. Environ Health Perspect.

[b15-ehp0115-000463] Fine BP, Vetrano T, Skurnick J, Ty A (1988). Blood pressure elevation in young dogs during low-level lead poisoning. Toxicol Appl Pharmacol.

[b16-ehp0115-000463] Gerhardsson L, Chettle DR, Englyst V, Nordberg GF, Nyhlin H, Scott MC (1992). Kidney effects in long-term exposed lead smelter workers. Br J Ind Med.

[b17-ehp0115-000463] Goldstein GW, Asbury AK, Diamond I (1974). Pathogenesis of lead encephalopathy. Uptake of lead and reaction of brain capillaries. Arch Neurol.

[b18-ehp0115-000463] Gonzalez-Cossio T, Peterson KE, Sanín L, Fishbein SE, Palazuelos E, Aro A (1997). Decrease in birth weight in relation to maternal bone lead burden. Pediatrics.

[b19-ehp0115-000463] Gomaa A, Hu H, Bellinger D, Schwartz J, Tsaih S, Gonzalez-Cossio T (2002). Maternal bone lead as an independent risk factor for fetal neurotoxicity: a prospective study. Pediatrics.

[b20-ehp0115-000463] Gonick HC, Ding Y, Bondy SC, Ni Z, Vaziri ND (1997). Lead-induced hypertension: interplay of nitric oxide and reactive oxygen species. Hypertension.

[b21-ehp0115-000463] Gulson BL, Jameson CW, Mahaffey KR, Mizon KJ, Patison N, Law A (1998). Relationship of lead in breast milk to lead in blood, urine, and diet of the infant and mother. Environ Health Perspect.

[b22-ehp0115-000463] Gulson BL, Mizon KJ, Korsch MJ, Mahaffey KR, Taylor AJ (2001). Dietary intakes of selected elements from longitudinal 6-day duplicate diets for pregnant and nonpregnant subjects and elemental concentrations of breast milk and infant formula. Environ Res.

[b23-ehp0115-000463] Hänninen H, Aitio A, Kovala T, Luukkonen R, Matikainen E, Mannelin T (1998). Occupational exposure to lead and neuropsychological dysfunction. Occup Environ Med.

[b24-ehp0115-000463] Harlan WR (1988). The relationship of blood lead levels to blood pressure in the U.S. population. Environ Health Perspect.

[b25-ehp0115-000463] Hernandez-Avila M, Peterson KE, Gonzalez-Cossio T, Sanin LH, Aro A, Schnaas L (2002). Effect of maternal bone lead on length and head circumference at birth. Arch Environ Health.

[b26-ehp0115-000463] Hertz-Picciotto I (2000). The evidence that lead increases the risk for spontaneous abortion. Am J Ind Med.

[b27-ehp0115-000463] Hipkins KL, Materna BL, Payne S, Kirsch L (2004). Family lead poisoning due to occupation. Clin Pediatr.

[b28-ehp0115-000463] Hofreuter DH, Catcott EJ, Keenan RG, Xintaras C (1961). The public health significance of atmospheric lead. Arch Environ Health.

[b29-ehp0115-000463] Hu H, Aro A, Payton M, Korrick S, Sparrow D, Weiss ST (1996). The relationship of bone and blood lead to hypertension. JAMA.

[b30-ehp0115-000463] Hu H, Shih R, Rothenberg S, Schwartz BS (2007). The epidemiology of lead toxicity in adults: measuring dose and consideration of other methodologic issues. Environ Health Perspect.

[b31-ehp0115-000463] Hu H, Téllez-Rojo MM, Bellinger D, Smith D, Ettinger AS, Lamadrid-Figueroa H (2006). Fetal lead exposure at each stage of pregnancy as a predictor of infant mental development. Environ Health Perspect.

[b32-ehp0115-000463] Janakiraman V, Hu H, Mercado-Garcia A, Hernandez-Avila M (2003). A randomized crossover trial of nocturnal calcium supplements to suppress bone resorption during pregnancy. Am J Prev Med.

[b33-ehp0115-000463] Korrick SA, Hunter DJ, Rotnitzky A, Hu H, Speizer FE (1999). Lead and hypertension in a sample of middle-aged women. Am J Public Health.

[b34-ehp0115-000463] Kosnett MJ 2001. Lead. In: Clinical Toxicology (Ford M, Delaney KA, Ling L, Erickson T, eds). St. Louis:WB Saunders, 723–736.

[b35-ehp0115-000463] Kosnett MJ 2005. Lead. In: Critical Care Toxicology (Brent J, Wallace KL, Burkhart KK, Phillips SD, Donovan JW, eds). Philadelphia:Elsevier Mosby, 821–836.

[b36-ehp0115-000463] Krieg EF, Chislip DW, Crespo CJ, Brightwell WS, Ehrenberg RL, Otto D (2005). The relationship between blood lead levels and neurobehavioral test performance in NHANES III and related occupational studies. Public Health Rep.

[b37-ehp0115-000463] Lilis R, Gavrilescu N, Nestorescu B, Dumitriu C, Roventa A (1968). Nephropathy in chronic lead poisoning. Br J Ind Med.

[b38-ehp0115-000463] Lindgren KN, Ford DP, Bleeker ML (2003). Pattern of blood lead levels over working lifetime and neuropsychological performance. Arch Environ Health.

[b39-ehp0115-000463] Lustberg M, Silbergeld E (2002). Blood lead levels and mortality. Arch Intern Med.

[b40-ehp0115-000463] Mahaffey KR, Annest JL, Roberts J, Murphy RS (1982). National estimates of blood lead levels: United States, 1976–1980. N Engl J Med.

[b41-ehp0115-000463] Mantere P, Hänninen H, Hernberg S, Luukkonen R (1984). A prospective follow-up study on psychological effects in workers exposed to low-levels of lead. Scand J Work Environ Health.

[b42-ehp0115-000463] Manton WI, Angle CR, Stanek KL, Reese YR, Kuehnemann TJ (2000). Acquisition and retention of lead by young children. Environ Res.

[b43-ehp0115-000463] Menke A, Muntner P, Batuman V, Silbergeld EK, Guallar E (2006). Blood lead below 0.48 μmol/L (10 μg/dL) and mortality among US adults. Circulation.

[b44-ehp0115-000463] Minot AS (1938). The physiological effects of small amounts of lead: an evaluation of the lead hazard of the average individual. Physiol Rev.

[b45-ehp0115-000463] Muldoon SB, Cauley JA, Kuller LH, Morrow L, Needleman HL, Scott J (1996). Effects of blood lead levels on cognitive function of older women. Neuroepidemiology.

[b46-ehp0115-000463] Muntner P, Vupputuri S, Coresh J, Batuman V (2003). Blood lead and chronic kidney disease in the general United States population: results from NHANES III. Kidney Int.

[b47-ehp0115-000463] Nash D, Magder L, Lustberg M, Sherwin R, Rubin R, Kaufmann R (2003). Blood lead, blood pressure, and hypertension in perimenopausal and postmenopausal women. JAMA.

[b48-ehp0115-000463] National Center for Health Statistics 1984. Blood lead levels for persons ages 6 months to 74 years. United States, 1976–1980. Vital and Health Statistics. Ser 11, no 233. Publ no (PHS) 84–1683. Washington, DC:National Center for Health Statistics. 6333758

[b49-ehp0115-000463] Nawrot TS, Thijs L, Den Hond EM, Roels HA, Staessen JA (2002). An epidemiological re-appraisal of the association between blood pressure and blood lead: a meta-analysis. J Hum Hypertens.

[b50-ehp0115-000463] Navas-Acien A, Guallar E, Silbergeld EK, Rothenberg SJ (2007). Lead exposure and cardiovascular disease—a systematic review. Environ Health Perspect.

[b51-ehp0115-000463] Occupational Lead Poisoning Prevention Program 2006. Common Jobs, Hobbies & Other Sources of Lead. Richmond, CA:California Department of Health Services, Occupational Health Branch. Available: http://www.dhs.ca.gov/ohb/olppp/leadsources.pdf [accessed 14 December 2006]

[b52-ehp0115-000463] OSHA (U.S. Occupational Safety and Health Administration) 2002. Safety and Health Topics: Lead. Compliance. Available: http://www.osha.gov/SLTC/lead/compliance.html [accessed 14 December 2006].

[b53-ehp0115-000463] OSHA (U.S. Occupational Safety and Health Administration) 2005. Blood Lead Laboratories Program Description and Background. Available: http://www.osha.gov/SLTC/bloodlead/program.html [accessed 14 December 2006].

[b54-ehp0115-000463] Parsons PJ, Reilly AA, Hussain A (1991). Observational study of erythrocyte portoporphyrin screening test for detecting low lead exposure in children; impact of lowering the blood lead action threshold. Clin Chem.

[b55-ehp0115-000463] Payton M, Hu H, Sparrow D, Weiss ST (1994). Low-level lead exposure and renal function in the Normative Aging Study. Am J Epidemiol.

[b56-ehp0115-000463] Payton M, Riggs KM, Spiro A, Weiss ST, Hu H (1998). Relations of bone and blood lead to cognitive function: the VA Normative Aging Study. Neurotoxicol Teratol.

[b57-ehp0115-000463] Piacitelli GM, Whelan EA, Ewers LM, Sieber WK (1995). Lead contamination in automobiles of lead-exposed bridgeworkers. Appl Occup Environ Hyg.

[b58-ehp0115-000463] Pocock SJ, Shaper AG, Ashby D, Delves HT, Clayton BE (1988). The relationship between blood lead, blood pressure, stroke, and heart attacks in middle-aged British men. Environ Health Perspect.

[b59-ehp0115-000463] Rabinowitz MB, Bellinger D, Leviton A, Needleman H, Schoenbaum S (1987). Pregnancy hypertension, blood pressure during labor, and blood lead levels. Hypertension.

[b60-ehp0115-000463] Rogan WJ, Dietrich KN, Ware JH, Docery DW, Salganik M, Radcliffe J (2001). The effects of chelation therapy with succimer on neuropsychological development in children exposed to lead. N Engl J Med.

[b61-ehp0115-000463] Roscoe RJ, Gittleman JL, Deddens JA, Petersen MR, Halperin WE (1999). Blood lead levels among children of lead-exposed workers: a meta-analysis. Am J Ind Med.

[b62-ehp0115-000463] Rothenberg SJ, Kondrashov V, Manalo M, Jiang J, Cuellar R, Garcia M (2002). Increases of hypertension and blood pressure during pregnancy with increased bone lead. Am J Epidemiol.

[b63-ehp0115-000463] Rothenberg SJ, Manalo M, Jiang J, Cuellar R, Reyes S, Sanchez M (1999a). Blood lead levels and blood pressure during pregnancy in South Central Los Angeles. Arch Environ Health.

[b64-ehp0115-000463] Rothenberg SJ, Schnaas L, Perroni E, Hernandez RN, Martinez S, Hernandez C (1999b). Pre- and postnatal lead effect on head circumference: a case for critical periods. Neurotoxicol Teratol.

[b65-ehp0115-000463] Sanín LH, González-Cossín T, Romieu I, Perterson KE, Ruíz S, Palazuelos E, Hernández-Avila M, Hu H (2001). Effect of maternal lead burden on infant weight and weight gain at one month of age among breastfed infants. Pediatrics.

[b66-ehp0115-000463] Schnaas L, Rothenberg SJ, Flores MF, Martinez S, Hernandez C, Osorio E (2006). Reduced intellectual development in children with prenatal lead exposure. Environ Health Perspect.

[b67-ehp0115-000463] Schober SE, Miral B, Graudbard BI, Brody DJ, Flegal KM (2006). Blood lead levels and death from all causes, cardiovascular disease, and cancer: results from the NHANES III mortality study. Environ Health Perspect.

[b68-ehp0115-000463] Schwartz BS, Hu H (2007). Adult lead exposure: time for change. Environ Health Perspect.

[b69-ehp0115-000463] Schwartz BS, Lee BK, Lee GS, Stewart WF, Lee SS, Hwang KY (2001). Association of blood lead, dimercaptosuccinic acid-chelatable lead, and tibia lead with neurobehavioral test scores in South Korean lead workers. Am J Epidemiol.

[b70-ehp0115-000463] Schwartz BS, Lee BK, Bandeen-Roche K, Stewart W, Bolla K, Links J (2005). Lead dose is associated with longitudinal decline in neurobehavioral test scores in South Korean lead workers. Epidemiology.

[b71-ehp0115-000463] Schwartz J (1988). The relationship between blood lead and blood pressure in NHANES II survey. Environ Health Perspect.

[b72-ehp0115-000463] Schwartz J (1995). Lead, blood pressure, and cardiovascular disease in men. Arch Environ Health.

[b73-ehp0115-000463] Sen D, Wolfson H, Dilworth M (2002). Lead exposure in scaffolders during refurbishment construction activity–an observational study. Occup Med.

[b74-ehp0115-000463] Shih RA, Glass TA, Bandeen-Roche K, Carlson MC, Bolla KI, Todd AC, Schwartz BS (2006). Environmental lead exposure and cognitive function in community-dwelling older adults. Neurology.

[b75-ehp0115-000463] Shih RA, Hu H, Weisskopf MG, Schwartz BS (2007). Cumulative lead dose and cognitive function in adults: a review of studies that measured both blood lead and bone lead. Environ Health Perspect.

[b76-ehp0115-000463] Sinks T, Jackson RJ (1999). International study finds breast milk free of significant lead contamination. Environ Health Perspect.

[b77-ehp0115-000463] Sowers MR, Scholl TO, Hall G, Jannausch ML, Kemp FW, Li X (2002). Lead in breast milk and maternal bone turnover. Am J Obstet Gynecol.

[b78-ehp0115-000463] Staeesen JA, Bulpitt CJ, Fagard R, Lauwerys RR, Roels H, Thijs L, Amery A (1994). Hypertension caused by low-level lead exposure: myth or fact?. J Cardiovasc Risk.

[b79-ehp0115-000463] Staessen JA, Lauwerys RR, Buchet JP, Bulpitt CJ, Rondia D, Vanrenterghem Y (1992). Impairment of renal function with increasing blood lead concentrations in the general population. The Cadmibel Study Group. N Engl J Med.

[b80-ehp0115-000463] Staessen JA, Roels H, Fagard R (1996). Lead exposure and conventional and ambulatory blood pressure: a prospective population study. PheeCad Investigators. JAMA.

[b81-ehp0115-000463] Stollery BT (1996). Reaction time changes in workers exposed to lead. Neurotoxicol Teratol.

[b82-ehp0115-000463] Tellez-Rojo MM, Hernandez-Avila M, Lamadrid-Figueroa H, Smith D, Hernandez-Cadena L, Mercado A (2004). Impact of bone lead and bone resorption on plasma and whole blood lead levels during pregnancy. Am J Epidemiol.

[b83-ehp0115-000463] Theppeang K, Schwartz BS, Lee BK, Lustberg ME, Silbergeld EK, Kelsey KT (2004). Associations of patella lead with polymorphisms in the vitamin D receptor, delta-aminolevulinic acid dehydratase and endothelial nitric oxide synthase genes. J Occup Environ Med.

[b84-ehp0115-000463] Tsaih SW, Korrick S, Schwartz J, Amarasiriwardena C, Aro A, Sparrow D (2004). Lead, diabetes, hypertension, and renal function: Normative Aging Study. Environ Health Perspect.

[b85-ehp0115-000463] U.S. EPA 2006. Air Quality Criteria for Lead (Final). Washington, DC:U.S. Environmental Protection Agency, National Center for Environmental Assessment. Available: http://cfpub.epa.gov/ncea/cfm/recordisplay.cfm?deid=158823 [accessed 14 December 2006].

[b86-ehp0115-000463] Vaziri ND (2002). Pathogenesis of lead-induced hypertension: role of oxidative stress. J Hypertens.

[b87-ehp0115-000463] Vupputuri S, He J, Munter P, Bazzano L, Whelton PK, Batuman V (2003). Blood lead level is associated with elevated blood pressure in blacks. Hypertension.

[b88-ehp0115-000463] Wasserman GA, Liu X, Popovac D, Factor-Litvak P, Kline J, Waternaux C (2000). The Yugoslavia prospective lead study: contributions of prenatal and postnatal lead exposure to early intelligence. Neurotoxicol Teratol.

[b89-ehp0115-000463] Weaver VM, Lee B-K, Ahn K-D, Lee G-S, Todd AC, Stewart WF (2003). Associations of lead biomarkers with renal function in Korean lead workers. Occup Environ Med.

[b90-ehp0115-000463] Weaver VM, Lee BK, Todd AC, Ahn KD, Shi W, Jaar BG (2006). Effect modification by δ-aminolevulinic acid dehydratase, vitamin D receptor, and nitric oxide sythase gene polymorphisms on associations between patella lead and renal function in lead workers. Environ Res.

[b91-ehp0115-000463] Wedeen RP, Mallik DK, Batuman V (1979). Detection and treatment of occupational lead nephropathy. Arch Intern Med.

[b92-ehp0115-000463] Weisskopf MG, Proctor SP, Wright RO, Schwartz J, Spiro A, Sparrow D, Nie H, Hu H (2007). Cumulative lead exposure and cognitive performance among elderly men. Epidemiology.

[b93-ehp0115-000463] Weisskopf MG, Wright RO, Schwartz J, Spiro A, Sparrow D, Aro A (2004). Cumulative lead exposure and prospective change in cognition among elderly men: the VA Normative Aging Study. Am J Epidemiol.

[b94-ehp0115-000463] Winker R, Barth A, Ponocny-Seliger E, Pilger A, Osterode W, Rudiger HW (2005). No cognitive deficits in men formerly exposed to lead. Wein Klin Wochenschr.

[b95-ehp0115-000463] Winker R, Ponocny-Seliger E, Rudiger HW, Barth A (2006). Lead exposure levels and duration of exposure absence predict neurobehavioral performance. Int Arch Occup Environ Health.

[b96-ehp0115-000463] Wright RO, Tsaih SW, Schwartz J, Spiro A, McDonald K, Weiss ST (2003). Lead exposure biomarkers and mini-mental status exam scores in older men. Epidemiology.

[b97-ehp0115-000463] Wu MT, Kelsey K, Schwartz J, Sparrow D, Weiss S, Hu H (2003). A δ-aminolevulinic acid dehydratase (ALAD) polymorphism may modify the relationship of low level lead exposure to uricemia and renal function: the Normative Aging Study. Environ Health Perspect.

